# Prevention of hospital-acquired thrombosis from a primary care perspective: a qualitative study

**DOI:** 10.3399/bjgp16X685693

**Published:** 2016-06-07

**Authors:** Ian Litchfield, David Fitzmaurice, Patricia Apenteng, Sian Harrison, Carl Heneghan, Alison Ward, Sheila Greenfield

**Affiliations:** Institute of Applied Health Research, College of Medical and Dental Sciences, University of Birmingham, Edgbaston.; Primary Care Clinical Sciences, College of Medical and Dental Sciences, University of Birmingham, Edgbaston.; College of Medical and Dental Sciences, University of Birmingham, Edgbaston.; Nuffield Department of Primary Care Health Sciences, University of Oxford, Oxford.; Nuffield Department of Primary Care Health Sciences, University of Oxford, Oxford.; Nuffield Department of Primary Care Health Sciences, University of Oxford, Oxford.; College of Medical and Dental Sciences, University of Birmingham, Edgbaston.

**Keywords:** prevention and control, primary health care, qualitative research, thrombosis

## Abstract

**Background:**

Although there is considerable risk for patients from hospital-acquired thrombosis (HAT), current systems for reducing this risk appear inefficient and have focused predominantly on secondary care, leaving the role of primary care underexplored, despite the onset of HAT often occurring post-discharge.

**Aim:**

To gain an understanding of the perspectives of primary care clinicians on their contribution to the prevention of HAT. Their current role, perceptions of patient awareness, the barriers to better care, and suggestions for how these may be overcome were discussed.

**Design and setting:**

Qualitative study using semi-structured interviews in Oxfordshire and South Birmingham, England.

**Method:**

Semi-structured telephone interviews with clinicians working at practices of a variety of size, socioeconomic status, and geographical location.

**Results:**

A number of factors that influenced the management of HAT emerged, including patient characteristics, a lack of clarity of responsibility, limited communication and poor coordination, and the constraints of limited practice resources. Suggestions for improving the current system include a broader role for primary care supported by appropriate training and the requisite funding.

**Conclusion:**

The role of primary care remains limited, despite being ideally positioned to either raise patient awareness before admission or support patient adherence to the thromboprophylaxis regimen prescribed in hospital. This situation may begin to be addressed by more robust lines of communication between secondary and primary care and by providing more consistent training for primary care staff. In turn, this relies on the allocation of appropriate funds to allow practices to meet the increased demand on their time and resources.

## INTRODUCTION

Hospital-acquired thrombosis (HAT) is a substantial healthcare problem resulting in significant mortality, morbidity, and economic cost.^1^^,^^2^ Recent estimates put the figures for hospital deaths from venous thromboembolism (VTE) in England and Wales in excess of 34 000 3 out of some 16 million admissions,^4^ although the introduction of the VTE risk assessment tool has led to a reduction in these numbers.^5^ It is a disorder that can occur across race, ethnicity, age group, and sex, with many of the known risk factors, such as advanced age, immobility, surgery, and obesity, on the increase. HAT can occur up to 90 days after admission,^6^ yet, to date, much of the focus on preventing HAT has fallen on the secondary care environment and there is little to no understanding of the role of primary care. However, a recent study that incorporated primary care data found that over 50% of deaths from VTE occurred after hospital discharge.^7^

This risk of developing HAT is influenced by the specific medical condition of the patient^8^ and thromboprophylaxis has been shown to reduce the risk of VTE by 75% in surgical patients^9^ and by around 50% in medical patients.^9^^,^^10^

**Figure 1.**
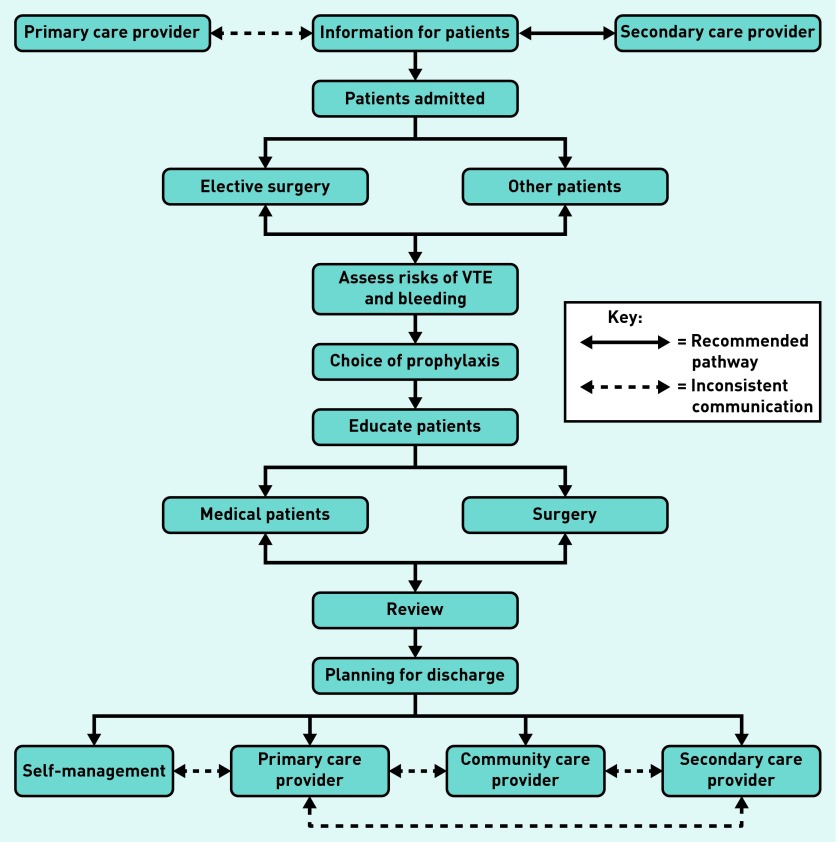
***Management of VTE risk in hospitalised patients (after NICE 2010).^11^ VTE = venous thromboembolism.***

Current UK guidelines for preventing HAT^11^ (Figure 1) recommend using the Department of Health’s risk assessment tool^12^ to inform the prescription of the appropriate thromboprophylaxis.^13^ The risk assessment tool uses factors, such as significant comorbidity, age, and pregnancy, alongside the risks associated with hospital admissions, such as reduced mobility for >3 days or undergoing surgery that lasts >60 minutes. The prophylaxis that is recommended consists of mechanical devices, such as antiembolism stockings, often used in combination with a pharmacological element including low molecular weight heparin (LMWH), sometimes prescribed for several months following surgery.^11^ Previous research abroad has indicated that non-adherence to guidelines is an issue for both physicians^14^ and patients.^15^^,^^16^ There is some evidence of similar issues of adherence among patients in the UK,^17^ with some reporting adherence to LMWHs as low as 23%.^18^ The guidelines also stipulate a supporting role for GPs, based on their notification of when patients are discharged and the prophylaxis prescribed. This type of communication between care settings is known to be problematic,^19^^–^23 leaving patients vulnerable to adverse events following discharge,^24^^–^29 and the role performed by primary care being unclear.

If primary care is to contribute more effectively to the prevention of HAT, then a better understanding of its current role and of the factors that influence this role is required. The ExPeKT study was designed to explore existing knowledge of thromboprophylaxis among patients, clinicians, and related staff in primary and secondary care, and other relevant organisations.^30^ Here the authors report on a qualitative exploration of the perspectives of primary care clinicians on the factors that influence HAT prevention, including potential barriers to improving current systems and how they may be overcome.

**How this fits in**

Large numbers of patients are affected by hospital-acquired thrombosis. There is a clear need to improve current mechanisms for managing the issue. Primary care can fulfil this need, although currently its role is poorly defined and it remains underutilised. The authors conducted a series of semi-structured interviews with primary care clinicians to explore perceptions of the current processes for preventing HAT across primary and secondary care. In doing so, ideas were gleaned on how the current management of HAT might be improved. Participants spoke of their limited role, both in educating patients and assessing the risk of HAT before admission, and the lack of contact with patients post-discharge. A number of reasons for this emerged, including a lack of clarity on the responsibility for patients, poor levels of communication, and, as a result, poor coordination of care between different settings. If a broader role for primary care is to be adopted, then there must be improved training for the relevant staff and the provision of appropriate resources.

## METHOD

The study sample was drawn from two former primary care trusts in Oxfordshire and South Birmingham. All 817 GPs and 583 practice nurses within the study area were sent a postal survey as part of the broader ExPeKT study and invited to participate in a semi-structured interview. From the 111 surveys that were returned, a total of 37 professionals confirmed they would be prepared to be interviewed. Following further contact by telephone, it was determined that, of these, three had retired and a further 20 were either unable to find a convenient time to take part or requested an online interview, which they failed to complete. A final total of 14 interviews took place: 12 GPs and two advanced nurse practitioners. Informed consent was obtained prior to conducting the interviews, which lasted between 10 and 50 minutes.

The study used semi-structured telephone interviews^31^ and a topic guide developed to explore clinicians’ awareness of hospital-associated VTE, their perceptions of the awareness of patients, and the role of primary care in managing this problem, including any limiting factors and ways in which current systems of managing the issue might be improved (see Box 1 for topic guide). The interviews were conducted by a research fellow experienced in qualitative research, recorded using a telephone recording adaptor with a digital recorder, and transcribed verbatim.

Box 1. **Topic guide for semi-structured telephone interviews**

- To what extent are GPs aware that hospital-acquired thrombosis (HAT) is a problem?
  – What is your awareness of existing guidelines?
- To what extent are patients aware of HAT?
  – Are there any characteristics of patients that affect this awareness?
  – Do they recognise symptoms?
- Where do you feel responsibility lies for preventing HAT?
- What is the role of primary care in managing HAT in the community?
  – Do you have contact with a patient either prior to admission or following discharge?
  – What are the factors that influence this patient contact?
- What are the factors that limit your role in managing HAT
  – What is the level of contact with other care providers?
  – What are the time and financial pressures?
  – Have you received any training for HAT risk assessment and management?
  – Do you feel that you receive adequate information from secondary care?
- How can the risk of HAT in the community be reduced?
  – Can primary care play a useful role?
  – What can facilitate any change in role?

### Analysis

Each transcript was read and the findings analysed by two of the authors, who agreed on themes and decided upon the coding framework. Transcripts were analysed using a framework analysis.^32^

## RESULTS

The sex of the participating clinicians are provided in Table 1, alongside a description of each practice, including the number of patients registered, Index of Multiple Deprivation ranking (IMD code),^33^ and an indication of rurality.^34^ The interviewed male and female GPs were from across eight practices. The practices were predominantly situated in urban environments; the IMD code varied from 4.29 to 39.69 and the number of patients from 3375 to 27 261. In addition, two advanced nurse practitioners at a large NHS community healthcare trust, which clinically manages people in their own homes to prevent an avoidable hospital admission, were interviewed.

**Table 1.** Characteristics of clinicians interviewed and their practices

**Clinician**	**Study practice**	**Sex**	**IMD code**	**Patient list**	**Urban/rural**
**GPs**					
GP01	Practice 1	Male	15.10	9595	A1 (Urban)
GP02	Practice 2	Male	39.69	9364	A1 (Urban)
GP03	Practice 3	Male	11.05	13 097	C1 (Urban)
GP04	Practice 3	Male	11.05	13 097	C1 (Urban)
GP05	Practice 4	Male	29.44	27 261	A1 (Urban)
GP06	Practice 5	Female	4.29	11 321	C1 (Urban)
GP07	Practice 6	Female	5.02	5917	E1 (Rural)
GP08	Practice 7	Female	10.08	3375	E1 (Rural)
GP09	Practice 8	Female	37.80	4115	C1 (Urban)
GP10	Practice 8	Male	37.80	4115	C1 (Urban)
GP11	Practice 8	Male	37.80	4115	C1 (Urban)
GP12	Practice 8	Male	37.80	4115	C1 (Urban)

**Nurse practitioners**					
NP01	Community healthcare trust 1	Male	31.70	n/a	A1 (Urban)
NP02	Community healthcare trust 1	Female	31.70	n/a	A1 (Urban)

IMD = Index of Multiple Deprivation.

The factors that influence the prevention and management of HAT in primary care are described here within five key themes: GP awareness, patient characteristics, designation of responsibility, communication across care settings, and logistical constraints. In discussing suggestions for the way in which the risk of HAT might be reduced, ideas emerged within two key themes: either clinical innovation or organisational innovation. The key themes and associated subthemes are described in Box 2.

Box 2. **Themes and subthemes**

**Table:** 

	**Influences on hospital-acquired thrombosis prevention in primary care**				**Suggestions for improving current systems**	
	
**GP awareness**	**Patient characteristics**	**Designation of responsibility**	**Coordination of care**	**Logistical constraints**	**Clinical innovation**	**Organisational innovation**
Current role Training	Awareness Clinical dependency	Secondary care Primary care	Communication with primary care and secondary care Communication with primary care and community care	Pre-admission risk assessment Increasing patient awareness Post-discharge appointments	Oral-based medication Software-based clinical support tool	Improved auditing Increased role of primary care Unified commissioning

### Influences on HAT prevention in primary care

#### GP awareness of HAT

The clinicians interviewed discussed their overall awareness of HAT and the nature of their specific role in its prevention. There appeared to be a general awareness of the risk of HAT to patients:

‘I’m aware that it’s becoming a huge problem because I know that they screen everybody now, pretty much everybody has to be on prophylaxis.’

(GP06)

‘I’m sure that the GPs are aware of it as a problem, yes.’

(NP02)

There appeared, however, little training specific to HAT other than that associated with the use of related medication:

‘I’ve probably not received official training along those lines, apart from warfarin, but no, no official training.’

(GP01)

Nor were several of those interviewed aware of the existing guidelines for reducing the risk of HAT, including the risk factors that would require extended prophylaxis following discharge:

‘There are hopefully protocols in place to prevent post-op VTE.’

(GP02)

‘Right now certainly I don’t know which operations do and don’t need extended prophylaxis.’

(GP03)

#### Patient characteristics: clinical dependency and patient awareness

Clinicians described how clinical dependency and patient education would influence the level of involvement of primary care providers.

A patient whom the practice recognises as being particularly vulnerable would be reviewed either prior to admission or following discharge:

‘We don’t often see them unless either there’s something that’s flagged up in pre-op assessments, or if they’ve got particular concerns. I mean, we wouldn’t routinely see someone, you know, before they go in for an operation.’

(GP02)

‘I think people who’ve had a prolonged admission or people who have multiple comorbidity or who are generally quite frail, you know, we might go and do a review post-discharge, particularly people on the Gold Standards Framework.’

(GP02)

Where patients were vulnerable, GPs would either administer prophylaxis or otherwise enlist the support of district nurses:

*‘Yes, we’re more than happy to give that* [Clexane^®^] *out to our patients — those patients who are elderly and are unable to administer it.’*

(GP01)

‘We get involved sometimes in arranging district nurses to administer extended courses of antithrombotics but it is very limited at the moment.’

(GP09)

‘We also get our district nurses to go out and give them their Clexane injections.’

(GP01)

The GPs described how some of the patients were vaguely aware of the issue, but not to the extent that they would recognise the symptoms:

‘I think they’re well aware that DVT involves getting a clot in your leg somewhere. I don’t think they’re too clued up about what the true symptoms are.’

(GP01)

None of those interviewed felt that the patients were appropriately informed. Some questioned the effectiveness of the communication of educational information:

‘I don’t think they’re educated when they go into hospital.’

(GP03)

‘They will always pretend that nobody has said anything, because they don’t understand a lot of it. They say, “Oh no, nobody’s ever said anything to me”, and you know right well they have. They often say, “I haven’t been told anything”, because they just don’t understand what’s being said.’

(NP01)

#### Designation of responsibility

Opinions varied on where responsibility for various aspects of HAT prevention should lie.

In considering educating patients, it was felt that the consultant within secondary care should bear responsibility:

‘If a hospital consultant is tabling somebody for surgery that is risky for DVT; they should be the one that is counselling the patient about DVT.’

(GP06)

There were various opinions on who was responsible for patients adhering to their HAT prophylaxis prescription:

‘A difficult one, I mean it’s been initiated in hospital and it’s prescribed in hospital, so I would guess in the current system, it would have to be the hospital that was responsible.’

(NP02)

‘I think once they’ve had their operation done, I think it’s a grey area, in terms of where the responsibility lies. Does it lie with consultants who’ve done the operation to make sure that they’ve sent patients home with prophylaxis, or whether it’s our job then to just make sure they are on prophylaxis when they come out?’

(GP01)

Others believed that, following discharge, the responsibility automatically falls on primary care, based on the assumption that patients had previously received the appropriate information:

‘Once they’re discharged on a 2-week course, it’s obviously the GP’s responsibility if they run into any problems. So as long as they’ve been advised what to look out for, then they would contact us if there are any problems.’

(GP02)

#### Communication with primary care, secondary care, and community care

GPs reported difficulties in coordinating care with colleagues in secondary and community-based care, primarily as a result of poor communication.

This poor communication appeared to be an issue, both before admission and following discharge. Clinicians reported that, though they would generally receive notification of admission, the detail it contained could vary:

‘Yes, we know they’re going in invariably, if it’s a planned admission … sometimes we know the date, sometimes we don’t know the date.’

(GP08)

The inconsistent quality of the discharge summary was also reported, as was the lack of information the practice received relating to extended prophylaxis:

‘That’s completely pot luck. Some discharge summaries are very good, they tell you the dose of Clexane that they want you to give and for how many weeks and what they’re treating for … and then, on the other hand, you just don’t really get any feedback at all.’

(GP01)

Another GP also noted the lack of precise information on extended prophylaxis:

‘Some of my patients have had, for example, a hip replacement and have had 35 days of injections; unless the patient tells you, you are not necessarily aware they are still taking it.’

(GP09)

One GP attributed the variation in the quality of the discharge summary to the inexperience of the author:

‘Well the problem is the hospital discharge notes are written by very junior staff, they’re writing them and they probably didn’t know what they were writing it for.’

(GP03)

One of the GPs interviewed reported the problems of liaising with district nurses over the care of discharged patients:

*‘The district nurse still comes in* [but] *it’s completely fragmented now. District nurses don’t work with you any more, they are in a separate team. They are employed by the hospitals now and communication is extremely poor.’*

(GP09)

#### Logistical constraints

Several of the GPs interviewed described how the pressure on resources in primary care precluded increased involvement in preventing HAT:

‘It’s not part of the core services of a GP and one can’t keep taking on sort of secondary care work without a funding stream.’

(GP08)

Another GP described how current demands on their time meant they were unwilling to assume responsibility for educating patients about the risks of HAT:

‘At the moment we are seriously swamped with other work we’ve already got from the hospital and it would need a nurse’s appointment for every patient going into hospital. So we would have to see them specifically to do this and so we absolutely, totally don’t want to take it on.’

(GP06)

There were also concerns voiced over the amount of time it would take to visit immobile patients following discharge:

‘It would require a lot of time … the patients don’t want to come in to the GP surgery when they’ve just had an operation so you’re talking about sending doctors out to people’s homes to go and talk to them about injecting low molecular weight heparin and preventing VTE.’

(GP02)

### Suggestions for improvement

The suggestions for improvement can be placed in one of two groups. The first, organisational innovations, consists of improved auditing, an increased and appropriately funded role for primary care, and unified commissioning of HAT.

The second group can be considered clinical innovations, namely clinical support tools and orally administered medication.

#### Improved auditing

One GP suggested that an important step was the systematic gathering of information on the time and cost issues of mismanaging HAT as a way of raising awareness and encouraging the appropriate investment:

‘I guess probably looking at the time and cost issues and putting that in front of the healthcare professionals and saying: “Look, this is something worthwhile doing because it does have financial and health costs if we don’t do it.”’

(GP01)

#### Increased role of primary care

It was acknowledged that an increased role for primary care could see benefits in a number of areas, including increased patient awareness and better coordination of care between primary and secondary care settings:

*‘Raising awareness of patients with planned admissions — that they ought to raise this issue* [HAT] *with the treating hospital — that would make a lot of sense.’*

(GP09)

GPs also felt that they could take a more proactive role in communicating with consultants following major surgery:

‘I think we as GPs should question discharges a bit more, especially after big operations. I think, at the moment, we do leave it in the hands of the consultants.’

(GP01)

The greater involvement of staff would require improved training of relevant staff:

‘Training, I think, would be good generally across all staff members, nurses, and doctors.’

(GP01)

#### Unified commissioning

It was also suggested that the commissioning could be unified and provision of prophylaxis should become the responsibility of a single organisation:

‘I would definitely commission the whole lot, not a week here and the rest prescribed by someone else.’

(GP09)

#### Clinical support tools

Software-based tools were mentioned as a means of supporting GPs to undertake any risk assessment:

‘Something like NHS Improvement should pick this up. Getting a risk assessment tool, a software tool, would be quite useful.’

(GP09)

#### Oral medication

Others felt that a more easily-administered medication would prove significant, reducing the need for clinician-mediated administration:

‘I mean, I’m looking forward to the time when oral anticoagulation will come and I know that that is available.’

(NP02)

## DISCUSSION

### Summary

Despite having the opportunity to actively reduce the occurrence of HAT, the current role of GPs and, more broadly, primary care, appears limited, whether in educating patients and assessing risk of HAT prior to admission, or in the management of patients on prophylaxis following discharge. The clinicians interviewed described a number of factors that influence prevention of HAT in primary care. These included limited awareness among GPs and poor coordination of care with colleagues in community or secondary care settings, exacerbated by a lack of clarity concerning their role and frequent inconsistencies in the quality and timing of communication between care settings.

A number of constructive suggestions did emerge to improve the current system, and there was a broad consensus that there was opportunity for an increased role for primary care both pre-admission and post-discharge. Those interviewed were equally clear that due to current logistical constraints, any extended role for primary care would require additional and targeted funding.

### Strengths and limitations

There is a growing understanding of the importance of managing HAT, though this is the first study to gain the perspectives of primary care providers. It cannot be commented on as to how representative these views are of the wider GP population; however, the practices represented a wide variety of IMD codes, list sizes, and geographical locations. Although telephone interviews were chosen over face-to-face interviews for practical reasons, short telephone interviews have been found to be equally as productive as short face-to-face interviews.^35^

Theoretical saturation was reached within the 14 interviews.^36^ The authors suggest that this comparatively small number could be explained by ‘consensus theory’, where ‘experts’ with shared knowledge about the topic under discussion are more likely to exhibit common values.^37^ The fact that so many GPs were too busy to be interviewed also supports the finding that the current demand for GP services limits the time available for undertaking additional activities.

### Comparison with existing literature

Patients were reported as being neither aware of the risk of HAT, nor how it might best be managed following discharge, despite recommendations to the contrary.^11^ Previous work indicates that appropriate patient education can improve outcomes and adherence to medication.^16^^,^^38^^,^^39^ Tools, such as enhanced medication plans, can improve information transfer and increase patient knowledge of individual drug treatment.^40^

The GPs interviewed also felt that this information might be better provided within the primary care environment. In hospital, patients can be flooded with information from doctors, frequently beyond their capacity to assimilate and memorise it,^41^ and, with shorter lengths of stay, ward staff are finding it harder to assess and meet the information needs of the patients,^42^ further inhibited by the complexity of the modern healthcare team.^43^ It has previously been suggested that greater responsibility for patient education should lie with primary care,^44^ where the quiet surroundings,^45^ managerial support,^46^^,^^47^ and the allocation of undisturbed time^44^ can facilitate improved communication.

Improving the coordination of HAT prevention between care settings would appear critical, considering the trend towards shorter hospital stays and increased delivery of care in the community.^48^^–^51 The coordination of care is key considering previous evidence of patients unprepared for their self-management role,^19^ and vulnerable to adverse events following discharge.^24^^–^29 However, the clinicians interviewed reported that any coordination was hindered by the fragmentation of their relationship with community care, and issues with the timeliness and content of the information they received from secondary care.

Of particular concern to many of the GPs interviewed was the quality of the discharge summary. These should be timely and contain information on newly prescribed medication or specific follow-up needs.^11^^,^^29^ However, many of the interviewed clinicians described them as late and frequently incomplete, reflecting previous evidence of GPs not routinely notified about patient admissions, discharges, or complications during the course of the hospital stay,^52^^–^55 and patients unable to access an appropriate healthcare practitioner in possession of their discharge summary.^20^^–^22 It was noted that summaries received from junior doctors were often poor, echoing previous research, which reported that junior doctors felt inadequately prepared for writing discharge summaries and needed improved training in the area.^56^ More robust systems of communication^57^^,^^58^ and increased involvement of informatics might benefit the production and dissemination of discharge summaries; both of these strategies have proven successful in other ‘high-risk’ circumstances.^59^ Another important aspect of the successful transition of patients is the mutually agreed transfer of responsibility from hospital to primary care provider;^29^ however, those interviewed offered conflicting opinions of where this responsibility should lie.

The National Institute for Health and Care Excellence (NICE) guidance is explicit in its recommendation for prompt and accurate communication with GPs, yet it would appear that this is not routinely followed. Though strategies have emerged that address HAT-specific barriers, such as continued education of junior doctors and giving greater prominence to medicated stockings on prescription charts,^60^^,^^61^ the means by which communication with primary care can be improved has yet to be explored.

It was acknowledged that primary care could support HAT prevention but it became clear that this was unlikely to happen without additional resources being available. Other suggestions to support the extended role for primary care advocated by some of those interviewed, such as improved training or the introduction of software-based clinical support, all have cost implications for an already stretched service.^62^ It was suggested that, in order to secure these funds, empirical evidence of the impact of HAT would help raise awareness of the issue and the financial implications of its mismanagement. In the absence of increased funding, the option remains to use existing resources more effectively. Recently, the use of pre-admission healthcare data has been successful in identifying high-risk cases of HAT,^63^ and it may be in the interim that this approach could help focus resources more precisely.

### Implications for practice

The number of patients with HAT is high and onset frequently occurs post-discharge. Despite this, the level of awareness among GPs varied and many of those interviewed agreed that improved training of GPs and other relevant staff is needed. With that in place, primary care staff would be better equipped to raise awareness of HAT in patients, undertake a potentially better informed risk assessment, and support vulnerable groups in adherence to the prescribed thromboprophylaxis.

There appeared to be a lack of clarity of what was expected from primary care. This included confusion about where the responsibility for preventing HAT lay, and when and how primary care providers might be involved. An improved definition of the role of primary care would be useful and is reliant on the provision of the appropriate training.

This better-defined role for primary care should be predicated on prompt and accurate communication of patient information between primary and secondary care. Currently, GPs reported reliance on second-hand information from patients. With access to the appropriate information, those patients at most risk from HAT can be more closely monitored and supported by GPs. Previous work has demonstrated the positive impact of a simple educational intervention for raising patient awareness on prophylaxis adherence following urology surgery.^18^ Piloting a similar intervention across a range of sites, involving a broader range of at-risk patient groups, should be considered.

There appears to be a useful role for primary care in the prevention of HAT. Gathering evidence of the impact of mismanaging HAT may encourage policymakers and commissioning bodies to prioritise the issue and provide the additional resources that would be required.
